# Improving Communication in Intensive Care Unit to Ward Transitions: Protocol for Multisite National Implementation of the ICU-PAUSE Handoff Tool

**DOI:** 10.2196/40918

**Published:** 2023-02-06

**Authors:** Elle Mizuki Fukui, Patrick G Lyons, Emily Harris, Emma K McCune, Juan C Rojas, Lekshmi Santhosh

**Affiliations:** 1 School of Medicine University of California, San Francisco San Francisco, CA United States; 2 Division of Pulmonary and Critical Care Medicine John T Milliken Department of Medicine Washington University School of Medicine in St. Louis St Louis, MO United States; 3 Healthcare Innovation Lab BJC HealthCare St Louis, MO United States; 4 Department of Medicine University of California, San Francisco San Francisco, CA United States; 5 Department of Internal Medicine Rush University Chicago, IL United States; 6 Division of Pulmonary, Critical Care, Allergy, and Sleep Medicine Department of Medicine University of California, San Francisco San Francisco, CA United States

**Keywords:** intensive care, medical error, communication, handoff, transfer, qualitative, sociotechnical theory, implementation science, medical education, communication tool, workflow, ICU, ward transition, ward transfer

## Abstract

**Background:**

The intensive care unit (ICU)–ward transfer poses a particularly high-risk period for patients. The period after transfer has been associated with adverse events and additional work for care teams related to miscommunication or omission of information. Standardized handoff processes have been found to reduce communication errors and adverse patient events in other clinical environments but are understudied at the ICU-ward interface. We previously developed an electronic ICU-ward transfer tool, ICU-PAUSE, which embeds the key elements and diagnostic reasoning to facilitate a safe transfer of care at ICU discharge.

**Objective:**

The aim of this study is to evaluate the implementation process of the ICU-PAUSE handoff tool across 10 academic medical centers, including the rate of adoption and acceptability, as perceived by clinical care teams.

**Methods:**

ICU-PAUSE will be implemented in the medical ICU across 10 academic hospitals, with each site customizing the tool to their institution’s needs. Our mixed methods study will include a combination of a chart review, quantitative surveys, and qualitative interviews. After a 90-day implementation period, we will conduct a retrospective chart review to evaluate the rate of uptake of ICU-PAUSE. We will also conduct postimplementation surveys of providers to assess perceptions of the tool and its impact on the frequency of communication errors and adverse events during ICU-ward transfers. Lastly, we will conduct semistructured interviews of faculty stakeholders with subsequent thematic analysis with the goal of identifying benefits and barriers in implementing and using ICU-PAUSE.

**Results:**

ICU-PAUSE was piloted in the medical ICU at Barnes-Jewish Hospital, the teaching hospital of Washington University School of Medicine in St. Louis, in 2019. As of July 2022, implementation of ICU-PAUSE is ongoing at 6 of 10 participating sites. Our results will be published in 2023.

**Conclusions:**

Our process of ICU-PAUSE implementation embeds each step of template design, uptake, and customization in the needs of users and key stakeholders. Here, we introduce our approach to evaluate its acceptability, usability, and impact on communication errors according to the tenets of sociotechnical theory. We anticipate that ICU-PAUSE will offer an effective handoff tool for the ICU-ward transition that can be generalized to other institutions.

**International Registered Report Identifier (IRRID):**

DERR1-10.2196/40918

## Introduction

### Background

The transition of a hospitalized patient from the intensive care unit (ICU) to the inpatient ward is a particularly high-risk period for the patient recovering from critical illness due to their medical complexity and often residual diagnostic uncertainty. Although transfer from the ICU is indicative of clinical improvement, this process encompasses changes in clinical environments, medical personnel, and reduced monitoring capacity. These complex patient transfers are vulnerable to provider miscommunication, which increases the risk of preventable medical errors and ICU readmissions [1-3]. The transition between medical teams is also distressing for patients and their families, particularly when there is poor communication with and among medical providers [1,4]. Although standardized practices and structured handoff tools reduce these errors and associated negative outcomes [5], these approaches are surprisingly understudied during this high-risk ICU-ward transition [6,7], which remains vulnerable to nonstandard and suboptimal practices [8].

A recent study of several teaching hospitals demonstrated high rates of communication errors—both those of commission and omission—and associated harms, including unplanned ICU readmissions, care delays, medication errors, missed results, and patient and family distress [9]. Residents compensated for suboptimal ICU-ward handoffs by recovering omitted information through the chart review, history-taking, duplicating previously completed tasks, or calling the ICU to clarify information; these inefficiencies and documentation burdens compete for trainee time that could otherwise be spent on patient care and education [2]. Prior studies demonstrate notable gaps in information that is reported by ICU providers and what is understood by ward providers, emphasizing the need for standardized written communication that accompanies the patient during the entire transfer process and mitigates the loss of essential clinical information [10].

These issues indicate the clear need for an evidence-based, standardized ICU-ward transfer tool. Given the variation in ICU systems across medical centers, successful implementation and sustainability of an ICU-ward handoff tool requires user-engaged design with potential institution-specific adaptations. Accordingly, we used human-centered design methods across multiple internal medicine residency programs to create and iteratively prototype a structured ICU-to-ward transfer tool embedded within the electronic health record (EHR) [11]. This template, ICU-PAUSE, comprises the following elements: brief ICU course and reason for admission (I), code status and goals of care (C), uncertainty of working diagnosis and diagnostic pause (U), pending tests (P), active consultants (A), deprescribing unnecessary medications and reviewing pertinent high-risk medications (U), summary of problems and uncompleted tasks (S), and exam at the time of transfer (E) [12].

The ICU-PAUSE handoff tool was piloted in the medical ICU at Barnes-Jewish Hospital (BJH) in 2019 and 2020, and will be implemented across 10 academic hospitals, with each site permitted to customize the tool to institution-specific needs. This mixed methods study will evaluate the feasibility and perceived impact of the novel EHR-embedded ICU-ward transfer tool, ICU-PAUSE, on communication failures and medical errors during the transfer of patients from the ICU to the inpatient ward.

### Study Aims and Objectives

This mixed methods study will evaluate the feasibility, acceptability, and perceived impact of a novel EHR-embedded transfer tool, ICU-PAUSE, on communication failures and medical errors during the ICU-ward transfer process. Our specific aims are as follows.

#### Aim 1

Evaluate the feasibility, acceptability, and adoption of implementing ICU-PAUSE into the ICU-ward physician transfer workflow in a diverse group of hospitals.

Informed by the Consolidated Framework for Implementation Research (CFIR) model and Sittig and Singh’s [13] sociotechnical theory [14], we will conduct quantitative surveys and semistructured interviews of faculty champions and resident trainee stakeholders from multiple institutions that have implemented ICU-PAUSE. Our thematic analysis will be based on dimensions of the sociotechnical model, which offers a framework to evaluate interdependent factors within complex ICU-ward workflows [13]. We aim to elicit perceptions of feasibility, informatics resource needs and constraints, barriers and facilitators to implementation, and cultural changes in safety surrounding ICU-ward handoffs. We will also perform a chart review of ICU-ward transfer notes to assess the frequency of ICU-PAUSE utilization as a measure of adoption.

#### Aim 2

Examine the perceived impact of ICU-PAUSE on communication failures and associated medical errors during ICU-ward transfers among internal medicine residents, ICU physicians, and ward physicians.

We will conduct quantitative surveys of postgraduate year 2 (PGY-2) and PGY-3 internal medicine residents, ICU attending physicians, and ward attending physicians (n=175) to assess the frequency of self-reported miscommunication events, medical errors, and adverse outcomes during ICU-ward handoffs after the implementation of ICU-PAUSE. We will compare these findings with previously collected preimplementation surveys [9].

This study will provide new evidence on potential best practices for implementation of a novel handoff tool in ICU discharge. ICU-PAUSE can potentially decrease communication failures in the ICU handoff process and be disseminated for adaptation to institution-specific needs, providing a valuable evidence-based transfer tool for ICU systems at other academic medical centers.

## Methods

### Context

**Figure 1:**
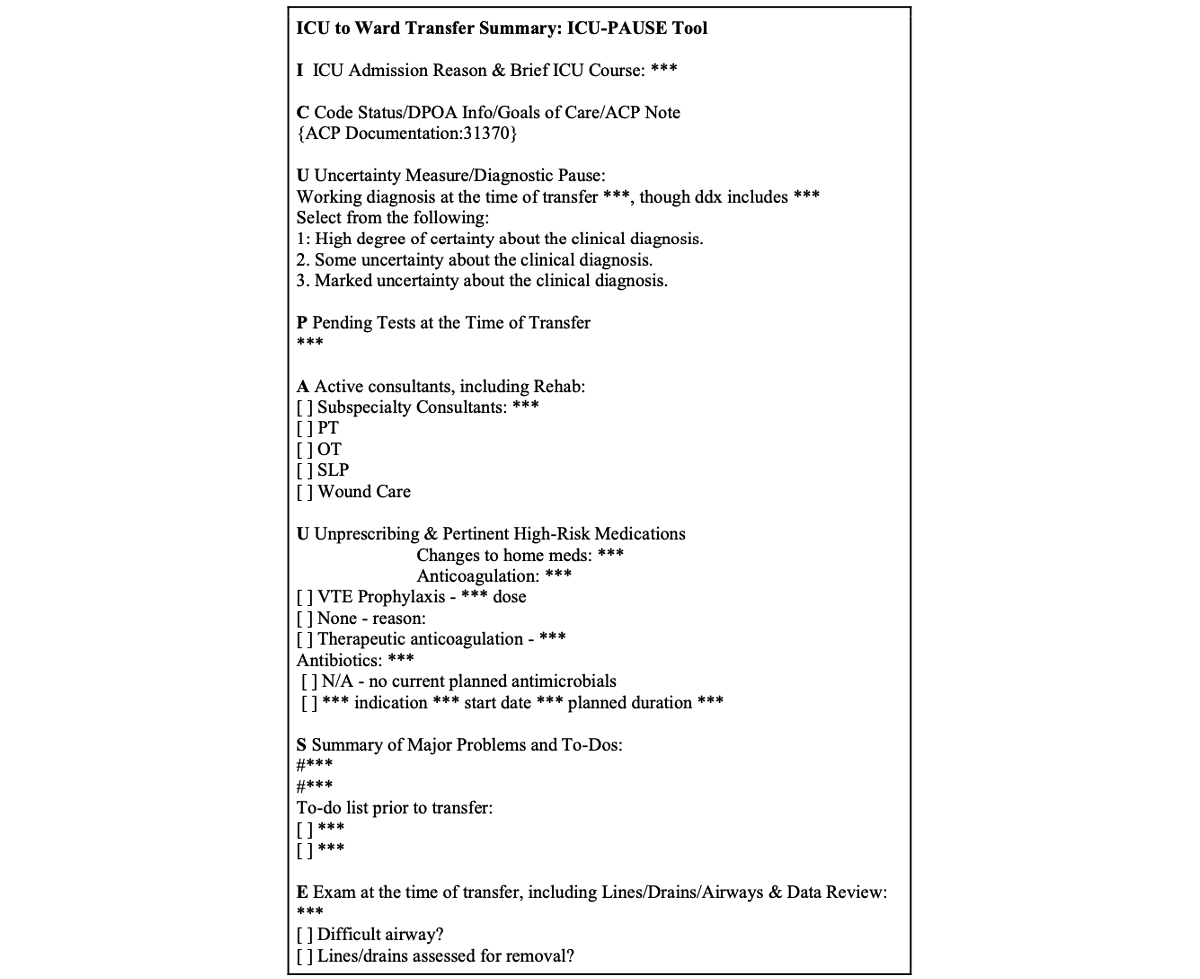
ICU-PAUSE Electronic Tool. ***: Free text placeholder; ACP: Advanced Care Planning; DPOA: Designated Power of Attorney; ICU: Intensive Care Unit; N/A: not applicable; OT: Occupational Therapy; PT: Physical Therapy; SLP: Speech and Language Pathology; VTE: Venous Thromboembolism Prophylaxis. Reproduced from Santhosh et al [12], which is published under Creative Commons Attribution Non-Commercial No Derivatives License 4.0 [15]).

Poor handoff communication between ICU and ward teams may result in unfavorable outcomes: studies have shown that standardization of communication during the handoff process reduces preventable medical errors [5]. We aimed to address the challenges that emerge during the ICU-ward transition period as a result of poor communication between ICU and ward providers by creating a standardized written handoff tool. The ICU-PAUSE handoff tool was developed between July 2019 and July 2020 and contains the key information to facilitate a safe transfer of care as identified through focus groups of residents (Figure 1) [12]. ICU-PAUSE will be integrated into the EHR and implemented into medical ICUs at the following sites between March and August 2022: Cedars-Sinai Medical Center, Kaiser Permanente Oakland Medical Center, Ohio State University, Penn State Health Milton S. Hershey Medical Center, Rutgers University, University of Chicago (UC), University of Iowa Hospitals and Clinics, University of Kentucky, and University of California-San Francisco (UCSF). Each site has a faculty champion, defined as an intensivist who has intimate knowledge of the ICU workflow and who will participate in an education session of the ICU-PAUSE tool to facilitate its implementation at their respective academic institutions. The ICU-PAUSE tool has been in use at our pilot site, BJH (the teaching hospital of Washington University School of Medicine in St. Louis [WUSM]) since late 2019 and is now the standard transfer summary documentation at this hospital.

### Study Design

This convergent mixed methods implementation study will use (1) quantitative surveys, (2) a retrospective chart review, and (3) qualitative semistructured interviews to explore the feasibility and perceptions of ICU-PAUSE after implementation at multiple sites [16]. Participants will include internal medicine residents, ICU attending physicians, and ward attending physicians in medical ICUs and inpatient wards across 10 academic medical centers. ICU residents and attendings who make the decision for patient transfer will compose their transfer note using the ICU-PAUSE tool, which will be received by residents and ward physicians on the inpatient wards team. Participants of the quantitative surveys will be recruited by email invitation to a web-based survey platform. Participants of the qualitative interviews will be contacted directly by the research team. For the quantitative arm of this study, we will survey residents, ICU physicians, and ward physicians at least 3 months after successful implementation of the ICU-PAUSE tool at their respective sites. A preimplementation survey of resident trainees was already conducted at UC, UCSF, and WUSM during the 2015-2018 academic years to evaluate the frequency of communication gaps and medical errors related to ICU-ward transfers [9]. We will re-administer this survey across all sites in the postimplementation period to compare survey responses from before and after the implementation of ICU-PAUSE. We will conduct a retrospective chart review of all ICU transfer notes within a 60-day period to evaluate the rate of tool uptake. For the qualitative component of this study, we will conduct pre- and postimplementation interviews of faculty champions who will facilitate implementation at their sites, as well as postimplementation interviews of residents and ICU physicians who have used the tool. The qualitative component of this study will complement our quantitative data by capturing stakeholder experiences surrounding the implementation process that may not be captured within the survey responses. This convergent mixed method approach will allow us to quantify the feasibility and acceptability of the ICU-PAUSE tool while also qualitatively assessing user experiences with the tool using the established implementation frameworks of CFIR and sociotechnical theory [13,14,17].

### Conceptual Framework

This study is informed by sociotechnical theory, which offers a paradigm for studying health information technology innovations [13]. The CFIR also provides the basis of our quantitative analysis to evaluate the implementation of ICU-PAUSE [14]. We will use these theoretical frameworks to evaluate ICU-PAUSE at both the user-technical interface and along constructs known to impact the success of implementation [18].

### Participants’ Inclusion and Exclusion Criteria

Aim 1 participants will include 1 faculty champion and 1 stakeholder (PGY-2 or PGY-3 internal medicine resident trainee or ICU attending physician) from each participating institution. Faculty champions have been identified a priori by word of mouth and will serve as key informants for identifying additional stakeholders for participation. Each faculty champion is an intensivist with an intimate understanding of the ICU-ward transfer workflow and will facilitate ICU-PAUSE implementation at their respective sites. The faculty champions from UC and UCSF, which we will refer to as lead sites, originally conceived of the ICU-PAUSE tool and will therefore be excluded from participation in any surveys or interviews. WUSM’s faculty champion at our pilot site was not directly involved in ICU-PAUSE development and will thus provide “early adopter” perspectives in this aim [19]. The remaining participating institutions will implement ICU-PAUSE between March and July 2022 and will be referred to as validation sites.

Aim 2 participants include all PGY-2 and PGY-3 internal medicine residents, ICU attending physicians, and ward attending physicians at all participating sites. PGY-1 residents will be excluded from the study because they infrequently hold primary responsibility for ICU-ward transfer communication at our institutions [9]. Key informants at each site will recruit survey participants, with no specific exclusion criteria.

### Intervention

There are 2 components to the intervention: an educational component and the ICU-PAUSE tool. In the educational component, the UCSF faculty champion conducts a 1-hour educational session on ICU-PAUSE implementation with the faculty champion from each participating site. The structured sessions entail discussing the rationale for the tool, describing how ICU-to-ward transitions are high-risk times for patients, walking through the template in detail, giving examples of template use, and answering questions. Once the educational component is completed, the ICU-PAUSE tool is made available as an EHR “dot-phrase” template that will be recreated at each site by the faculty champion (Figure 1). Local champions further the dissemination at the local site through a combination of educational sessions and flyers/handouts provided by the central ICU-PAUSE team. Adaptations will be noted and codified using the Framework for Reporting Adaptations and Modifications-Expanded (FRAME) method of tracking adaptations in our postimplementation interviews [20].

### Outcomes

The primary outcome in aim 1 will be the proportion of ICU-ward transfers for which a transfer note is written using the ICU-PAUSE template. Secondary outcomes will be perceptions of acceptability, appropriateness, and feasibility of ICU-PAUSE (as measured by the Acceptability of Intervention Measure [AIM], Intervention Appropriateness Measure [IAM], and Feasibility of Intervention Measure [FIM]) [21], perceived resource needs or constraints, and barriers or facilitators to implementation.

In aim 2, the primary outcome will be self-reported communication failures, the frequency of which will be compared with baseline preimplementation surveys. Secondary outcomes will include the perceived frequency of medical errors and self-reported time spent on tasks related to ICU-ward transfers.

### Data Collection

We conducted preimplementation semistructured interviews (n=7) and surveys (n=7) with the faculty champion from each validation site in October and November 2021.

In aim 1, we will re-administer the preimplementation survey to collect postimplementation responses from the same cohort of faculty champions. Survey questions are based on AIM, IAM, and FIM outcome measures to assess the acceptability, appropriateness, and feasibility of the ICU-PAUSE implementation along a 5-point Likert scale (Table 1) [21]. Surveys will be collected using Qualtrics web-based software.

Concurrently, we will conduct a retrospective chart review of patients (n=300) who were transferred from the ICU to inpatient wards between August and October 2022 across all sites. Outcome variables on patient demographics, provider demographics, and note template type will be extracted from ICU-ward transfer notes (Table 2). The adapted ICU-PAUSE template indicates use of the ICU-PAUSE tool with user modifications (Figure 1), whereas the non–ICU-PAUSE template refers to an ICU transfer note that uses any other pre-existing note template. Free text refers to a transfer note written without the use of any template. Each faculty champion will conduct a chart review at their respective institutions. Two co-investigators will independently review and compare a subset of 10 charts to ensure consistency in data collection.

Finally, we will repeat 45-minute semistructured interviews with each faculty champion, along with one additional internal medicine resident or ICU attending physician, for a total of 2 interviewees from each institution (n=14). The postimplementation interviews with the faculty champion will be used for comparison against the preimplementation interviews conducted with the same individual. Interviews with residents and ICU physicians who were not involved in the implementation process will provide an unbiased perspective on barriers or facilitators of ICU-PAUSE implementation. All interviews will be conducted via videoconference by the faculty champion at UCSF [22]. The interview guide was previously developed based on sociotechnical theory and will explore the following key constructs (Table 3): perceptions of usefulness, resource needs and constraints, barriers and facilitators to implementation, culture of safety, and adaptations [13,23]. Interviews will capture any adaptations to the ICU-PAUSE tool based on the FRAME method, such as the timing, participants, and nature of each modification [20]. All interviews will be audiorecorded with participant consent and transcribed verbatim.

In aim 2, we will conduct a postimplementation survey across all 10 sites to assess the frequency of communication failures, handoff errors (eg, rehabilitation needs and intravenous access), and time spent on tasks related to ICU-ward transfers (Table 4). The survey is identical in content to a preimplementation survey of internal medicine residents conducted at BJH, UC, and UCSF in our prior study, which characterized types of miscommunication errors in ICU-ward handoffs [9]. The survey will be administered to house officers, ICU physicians, and receiving ward physicians (n=175). The sample size is based on our work with the preimplementation cohort, and we will aim for at least 30% respondents at each site for the postimplementation surveys [9].

All faculty champions have connections with the internal medicine residencies and will similarly facilitate survey recruitment of residents and attendings at their respective sites. From August to October 2022, we will collaborate with chief residents to recruit PGY-2 and PGY-3 residents who are currently rotating on ICU. As residents rotate through ICU at different months of the academic year, we will email residents to complete the anonymous Qualtrics survey at the conclusion of their ICU rotation. Two follow-up emails will be sent 1 week later and 2 weeks later to maximize the response rate. The faculty champion will also email all ICU and ward attending physicians to complete the Qualtrics survey.

**Table 1:** Survey measures of faculty champions and stakeholders.

Survey components^a^	Examples of survey items
Acceptability of ICU^b^-PAUSE intervention	The ICU-PAUSE implementation strategy meets my approval The ICU-PAUSE implementation strategy is appealing to me
Appropriateness of ICU-PAUSE intervention	The ICU-PAUSE implementation strategy seems applicable The ICU-PAUSE implementation strategy seems suitable
Feasibility of ICU-PAUSE intervention	The ICU-PAUSE implementation strategy seems implementable The ICU-PAUSE implementation strategy seems possible

^a^Each survey component is graded along the Likert scale (on a scale of 1 to 5): strongly disagree (1), disagree (2), neither agree nor disagree (3), agree (4), strongly agree (5).

^b^ICU: intensive care unit.

**Table 2:** Chart review data extraction from ICU^a^-ward transfer notes.

Outcome variables	Items
Patient demographics	Age Sex/gender identity Race
Provider demographics	Resident Fellow ICU attending physician
Transfer note template	ICU-PAUSE template Adapted ICU-PAUSE template Non–ICU-PAUSE template Free text

^a^ICU: intensive care unit.

**Table 3:** Postimplementation semistructured interview measures.

Key concepts	Examples of interview questions
Background and current activities	What are the current efforts related to how handoffs occur when patients are being transferred from the ICU^a^ to the ward?
Perceptions of usefulness	How is the ICU-PAUSE framework helpful to you in improving the transition of care from the ICU to the ward?
Resource needs and constraints	What resources, personnel, informatics resources, etc. were necessary for you to implement the ICU-PAUSE framework and make this process actionable?
Barriers and facilitators	What were challenges for you to implement and meaningfully use the ICU-PAUSE framework? What might be the methods for measuring success?
Culture	How did the ICU-PAUSE framework change ideas of safety surrounding transition of care from the ICU to the ward?
Adaptations	Were there adaptations or modifications to the ICU-PAUSE framework at your institution? When did the modification occur? Were adaptations planned? Who participated in the decision to modify?

^a^ICU: intensive care unit.

**Table 4:** Survey measures of internal medicine residents, ICU^a^ physicians, and ward physicians [9].

Survey measures	Survey items
Provider demographics	Role (PGY-2^b^ resident, PGY-3 resident, ICU fellow, ICU attending physician, or ward attending physician) Age Gender identity Geographic region (Northeast, Midwest, South, West)
Frequency of omission or miscommunication of information in ICU-ward transfer note^c^	Rehabilitation (physical therapy/occupational therapy) needs Intravenous access and other indwelling hardware Risk assessment for ICU readmission Pending results Nutrition (per oral status, diet orders) Intravenous fluids Antibiotics Pain medications Insulin needs Venous thromboembolism prophylaxis and anticoagulation Vital signs Oxygen needs Mental status Delirium concerns Goals of care Health care decision-maker information
Frequency of adverse outcomes as a result of ICU-ward handoff^c^	Missed results Medication errors ICU readmission Rapid response activation Delayed discharge Patient lost in hospital Upset family Patient death
Time spent performing tasks related to ICU-ward handoff^d^	Writing transfer note Conducting handoff Chart review and order placement after handoff Repeating previously completed patient tasks Recovering information that should have been delivered at handoff Receiving handoff on an ICU patient who does not ultimately transfer

^a^ICU: intensive care unit.

^b^PGY: postgraduate year.

^c^Likert scale: Never (1), Rarely (<5 times/year) (2), Sometimes (~2 times/month) (3), Often (at least once weekly) (4), Always (nearly every handoff) (5).

^d^Likert scale: <15 minutes (1), 15-30 minutes (2), 30-60 minutes (3), 60-90 minutes (4), >90 minutes (5).

### Sample Size Considerations

As per best practices in human-centered design and qualitative inquiry, we have designed this study to include a sufficient number of stakeholders to capture essential design information and attain thematic saturation [24]. At the end of each interview, we will ask stakeholders if they have other stakeholder interviewees to suggest in a snowball-sampling style methodology to ensure that we have had the opportunity to hear new ideas from multiple participants.

### Analysis

For quantitative data, we will use descriptive statistics including frequencies, proportions, and measures of central tendency to compare pre- and postimplementation uptake, acceptability, and feasibility of ICU-PAUSE (Tables 1 and 2). We will also compare pre- and postimplementation surveys of self-reported communication failures, handoff errors, and time spent on ICU-ward transfers (Table 3) [9]. Pre- and postimplementation survey data will be compared using paired *t* tests. We will also evaluate for nonresponse bias by comparing the demographic data and survey responses of early respondents and late respondents, under the assumption that late respondents are similar to nonrespondents [25]. Early respondents are defined as participants who complete the survey within the first 2 weeks, whereas late respondents are those who complete the survey after a third reminder email is sent at 2 weeks [26]. Data analysis will be performed with Stata 17; *P* values less than .05 will be considered statistically significant.

We will analyze deidentified interview transcripts according to a deductive approach and perform thematic analysis independently on transcripts of preimplementation interviews and postimplementation interviews [27]. A coding framework rooted in the 8 dimensions of sociotechnical theory has been developed along the following codes: hardware and software, clinical content, human-computer interface, people, workflow and communication, internal organizational features, and external rules and regulations [13]. Along with the codes derived deductively from sociotechnical theory, we will include any codes that emerge inductively from the data in our final codebook. Two independent investigators will code the transcripts using Dedoose software (Dedoose). We will compare and resolve any coding discrepancies through an iterative process to achieve final consensus. Coded excerpts of the transcripts will be compiled to identify salient themes related to before and after ICU-PAUSE implementation.

### Researcher Reflexivity

The primary investigator of this study is a stakeholder in the development of ICU-PAUSE and will be conducting the faculty champion interviews. Two medical student co-investigators will observe the interviews for any bias, and we will regularly meet to discuss how personal or professional biases may impact the interviews and qualitative analysis during our deductive process.

### Ethical Considerations

We have received exemption by the institutional review board (IRB) to conduct the survey and interview arms of this study. We will apply for new IRB approval before conducting the chart review. Qualitative and quantitative data will be deidentified to ensure confidentiality. Informed consent for interview participation will be obtained verbally, whereas informed consent for survey participation will be embedded into the Qualtrics survey. Given that the outcome of ICU-PAUSE implementation impacts patient care and safety, we will cease implementation if responses reveal compromised patient safety.

## Results

We have already established proof of concept with the execution of ICU-PAUSE at our pilot site, BJH, in late 2019. As of July 2022, implementation of ICU-PAUSE is ongoing at all lead sites and validation sites. We intend to complete the study in mid-2023 and publish our results in the second half of 2023. We expect to identify the factors that facilitate and impede implementation of ICU-PAUSE, as well as a reduction in the frequency of communication failures related to ICU-ward transfers.

## Discussion

Currently available handoff tools [5] are informed by external resources and conceptual models [28] and lack the necessary elements to facilitate a safe transfer of information at the ICU-ward interface. These elements were identified and incorporated into our user-engaged creation of ICU-PAUSE, a novel clinical template specific to the ICU-ward transition [12]. Here, we aim to evaluate its acceptability, usability, and impact on communication errors using CFIR and sociotechnical theory [13,14]. Our work is innovative by centering the iterative process of template design [12], uptake, and customization in the hands of users who will implement ICU-PAUSE according to their institution’s needs. We anticipate that ICU-PAUSE will offer an acceptable and effective handoff tool that will reduce the frequency of communication failures during the ICU-ward transition.

We hypothesize that ICU-PAUSE will be used in at least 70% of ICU-ward transfer notes [5]. We expect to gain insight into the feasibility of ICU-PAUSE implementation, barriers or facilitators to uptake, any impact on the culture of safety surrounding ICU discharge, and adaptations made to the ICU-PAUSE tool. Our qualitative data will be interpreted according to sociotechnical theory, which provides a multidimensional framework to evaluate the process of ICU-PAUSE implementation within a complex health care system [13]. This interpretation will allow us to identify adaptations and potential problems in all domains, which is pivotal to the success of the intervention. We recognize that user interaction, the EHR interface, institutional workflows, and regulatory policies are all dynamic and interdependent. House officers represent the direct beneficiaries of ICU-PAUSE, whereas faculty stakeholders possess insight into internal institutional procedures and necessary adaptations to facilitate implementation. By interviewing both groups, we aim to capture a continuum of information that ranges from individual user experiences to overarching external regulations that influence the implementation process. ICU-PAUSE also seeks to address the need for brevity, clarity, and deliberate acknowledgment of diagnostic uncertainty in the ICU transfer summary [12]. With respect to our postimplementation surveys of house officers, ICU physicians, and ward physicians, we anticipate that ICU-PAUSE will reduce the frequency of communication errors, adverse outcomes, and time spent verifying information compared with preimplementation measures [9]. Thus, we hypothesize that the use of a structured communication ICU-PAUSE tool at transitions of care from the ICU to the ward will lead to a reduction in communication-related adverse events (frequency of omission or miscommunication of information in ICU-ward transfer notes) and no change or improvements in major adverse events (eg, delayed discharge, patient death, and ICU readmission).

The strengths of our work include the implementation of ICU-PAUSE at geographically diverse hospital systems, which will increase generalizability. The ability of each study site to customize ICU-PAUSE may also lead to higher uptake by providing a tool that meets the unique needs of each institution. We will include multiple perspectives by including house officers, ICU attending physicians, and faculty stakeholders. Our pre- and postimplementation data sets will allow us to compare inefficiencies and communication failures in the transfer process and determine whether they were remedied by ICU-PAUSE.

A notable limitation is the inconsistency of our postimplementation survey cohort of internal medicine trainees and attendings, which will not be identical to the preimplementation cohort from our prior study [9]. However, this is a known limitation of medical education research with resident trainees, and we will control for unpaired groups in our statistical analysis. We presume that residency training programs have remained consistent over the past several years during which the initial survey data were collected. There may also be selection bias by interviewing faculty champions who may hold personal or professional interests in the success of ICU-PAUSE. We also acknowledge that this study excludes the perspectives of other stakeholders including other specialty practitioners, nursing staff, and patients. We hope to build upon this work with further studies that will include patient and nursing input to gather further stakeholder input in this internal process. Lastly, this study will be conducted at medical lCUs at academic medical centers that may preclude generalizability to specialty ICUs. We considered using patient satisfaction data as a secondary outcome; however, as most patient satisfaction data surveys are collected after discharge, it is extremely challenging to distinguish patient satisfaction data related to the ICU stay or ICU transfer process alone rather than the entire hospitalization.

We intend to disseminate the future results of our multicenter study in a peer-reviewed journal to enable other health professionals to customize ICU-PAUSE to their own institutions and ICU systems. We also recognize the potential for systematic bias with any health technology intervention, and our future research will examine for any patterns of demographic or diagnostic bias in the use of ICU-PAUSE.

## Abbreviations

AIM: Acceptability of Intervention Measure
BJH: Barnes-Jewish Hospital
CFIR: Consolidated Framework for Implementation Research
EHR: electronic health record
FIM: Feasibility of Intervention Measure
FRAME: Framework for Reporting Adaptations and Modifications-Expanded
IAM: Intervention Appropriateness Measure
ICU: intensive care unit
IRB: institutional review board
PGY: postgraduate year
UC: University of Chicago
UCSF: University of California-San Francisco
WUSM: Washington University School of Medicine in St. Louis

## Data Availability

Transcriptions of deidentified postimplementation interviews will not be available publicly as they could easily be identified and deanonymize a specific site.
